# Perceived characteristics of life situations during the COVID-19 pandemic

**DOI:** 10.1192/j.eurpsy.2021.1786

**Published:** 2021-08-13

**Authors:** E. Bityutskaya, A. Kheruvimova, T. Mirzamedova, A. Skvortsova, A. Spitsyna, N. Lebedeva

**Affiliations:** 1 Faculty Of Psychology, Lomonosov Moscow State University, Moscow, Russian Federation; 2 Diagnostics Department, Moscow Metropolitan Governance University, Moscow, Russian Federation

**Keywords:** goal, COVID-19, perceived life situation, change

## Abstract

**Introduction:**

The COVID-19 pandemic situation is seen as an intense stressor. However, people process it differently.

**Objectives:**

This study aims to examine the connection between life situation perception and the desirability of life changes the pandemic caused.

**Methods:**

Adult participants (n=144; 01.04.2020–01.06.2020) answered open-ended questions about their current life situation experience and rated the desirability of life changes on a 10-point scale (see table 1). Content analysis and Pearson’s χ^2^ criterion were used.

**Table:** 

Table 1									
**Changes’ desirability**									
-5	-4	-3	-2	-1	+1	+2	+3	+4	+5
Group 1		Group 2			Group 3			Group 4	
Rejecting changes					Accepting changes

**Results:**

We annotated the participants’ responses. The content of life situations was categorized into restrictions, losses, difficulties (negative responses), acquisitions (positive responses), neutral, and ambivalent responses. Life goals were categorized into an approach to the desired outcome, avoidance of hassles, preservation of status quo, self-development, and return to prepandemic life. χ2 analysis confirms the differences between content-types and goal-types categories in 4 groups of participants: χ2(15)=43.62; p=0.002 (content); χ2(12)=27.23; p=0.01 (goals). The desirability of changes was positively linked with the ambivalent responses and responses containing self-development goals or approach-to-desired-outcome goals; and was negatively linked with the responses containing restriction-type situations and avoidance goals. Only the respondents accepting changes reported acquisitions; only the respondents rejecting changes reported a return to prepandemic life goals.

**Conclusions:**

Perceived characteristics of life situations are closely connected with the desirability of life changes during the pandemic. Funding: The reported study was funded by RFBR, project number 20-013-00838.

**Disclosure:**

No significant relationships.

